# Lives Transformed—The Experiences of Significant Others Supporting Patients With Severe Burn Injury: A Narrative Inquiry

**DOI:** 10.1111/jocn.70091

**Published:** 2025-09-02

**Authors:** Elizabeth Flannery, Kath Peters, Gillian Murphy, Elizabeth Halcomb, Lucie M. Ramjan

**Affiliations:** ^1^ School of Nursing and Midwifery Western Sydney University Penrith New South Wales Australia; ^2^ School of Nursing, Faculty of Science, Medicine & Health University of Wollongong Wollongong New South Wales Australia

**Keywords:** ICU, narrative inquiry, qualitative, severe burn injury, significant other, support, trauma

## Abstract

**Aim:**

To explore the experiences of significant others of patients with severe burn injury in the intensive care unit. Specifically, how severe burn injury impacted the significant other and their role within their loved one's life.

**Design:**

This qualitative study employed a Narrative Inquiry approach.

**Methods:**

Interviews were undertaken during 2021–2022 with 17 participants who were the significant others of a patient with severe burn injury in the Intensive Care Unit. Recruitment occurred in New South Wales, Australia, from two tertiary hospitals providing care for people with major burns. A narrative inquiry approach was utilised, capturing stories through semi‐structured interviews.

**Results:**

Significant others experienced necessary changes in their life in response to the catastrophe. These included advocating, being present and ensuring their loved one's needs were met, while often neglecting themselves. Significant others contemplated their future as a carer to their loved one with severe burn injury, and adjusting their own career, finances and lifestyle, often as a long‐term measure. The shifting of their role to carer ultimately transformed and redefined their relationships and lives.

**Conclusion:**

Significant others endure immense trauma when a loved one sustains a severe burn injury. They require support but prioritise the patient by virtue of their critical illness. The life of the significant other is changed as they take on the role of carer and provide support. It is, therefore, imperative that the support needs of significant others are recognised, understood and addressed to ensure their well‐being while processing the trauma.

**Implications for Practice:**

With increased understanding of the significant others' experiences, healthcare providers can adopt a consultative approach, where roles and boundaries can be clearly identified. Through this process, healthcare providers can strengthen rapport and provide targeted support for significant others, as they navigate this traumatic life‐altering event.

**Patient or Public Contribution:**

No patient or public contribution.

**Summary**

- What does this paper contribute to the wider global community?
  ○ Insights into the experiences of significant others of patients with severe burn injury allow nurses and other healthcare providers to align services to meet their needs.
  ○ Significant others are called upon to undertake new roles within the life of the patient with severe burn injury, which can bring permanent changes to their own life, career and relationships. Nurses and other healthcare providers need to take a collaborative approach to care planning and include significant others in this planning process so the support provided strengthens them in their capacity to undertake a carer role.
  ○ Not all significant others are able to undertake a carer role, due to career, parenting and lifestyle commitments. Nurses and other healthcare providers need to consider the complexities of becoming a carer and that support services need to match the needs of the significant other and patient with severe burn injury.
  ○ Significant others tend to focus on the support needs of the patient with severe burn injury, while neglecting their own basic needs. Nurses and other healthcare providers can address the significant others' needs with an assumption that support is required to ensure their needs are met.

## 1 Introduction

Significant others of Intensive Care Unit (ICU) patients with severe burn injury are pivotal to patient well‐being and survival (Bäckström et al. 2019). They are often required to engage in shared decision making with healthcare providers, including those around treatment options and end of life, based on their knowledge of the patient's wishes (Heidari Gorji et al. 2023). This immense responsibility occurs at a time of intense trauma, invoked by the severity of their loved one's burn injury and admission to ICU, where they face potential disability, disfigurement and survival that is uncertain (Bäckström et al. 2019). These factors place the significant other at high risk of psychological impacts such as anxiety and depression or post‐traumatic stress disorder, with the impact often being long‐term, affecting all aspects of their life, including their future relationship with the patient (Bäckström et al. 2019; Flannery et al. 2024). It is essential that significant others are supported in processing the trauma and coping with the changes to their role.

## 2 Background

Substantial research has been undertaken into the impact of severe burn injury on the life of the patient, yet there remains a paucity of literature on the experiences of the significant other in the acute phase and the subsequent long‐term impact on their life (Bäckström et al. 2019; Flannery, Halcomb, et al. 2022). In their scoping review, Bayuo and Wong (2021) note that there is limited research on significant others'/family members' concerns and long‐term issues related to burn injury overall. During the initial trauma of a severe burn injury and subsequent ICU admission, significant others are confronted with a sudden life‐altering event, which is often incomprehensible and destabilises their sense of control (Flannery et al. 2024; Tejero‐Aranguren et al. 2024). They are confronted with the prospect that the patient may die as a result of their injury (Heidari Gorji et al. 2023). Further, they are faced with fears related to their loved ones' psychological well‐being and their ability to cope with painful procedures such as wound dressings. In the long term, fears may include considering a life where their loved one experiences disability or disfigurement and is dependent on the significant other as a carer (Bäckström et al. 2019; Knol et al. 2020). Gullick et al. (2014) described the post‐burn relationship between significant others and patients as being encapsulated in a ‘trauma bubble’. This shift in the previous role of the significant other is necessary to contain the trauma and protect other family and friends from the negative experience (Gullick et al. 2014). While the ‘trauma bubble’ serves to protect the patient's privacy and protect others from the trauma, it also serves to isolate the significant other from seeking emotional support from family and friends (Gullick et al. 2014). While dated, this research demonstrates aspects of the significant others' experiences, which have shifted from the acute trauma and fears to adopting longer‐term coping responses.

The long‐term support and rehabilitation required in severe burn injury often has a major impact on the life of the significant other and their role within the patient's life (Johnson et al. 2016; Sundara 2014). Tejero‐Aranguren et al. (2024) identifies there is a huge burden for significant others of ICU patients who survive, referred to as the ‘forgotten caregivers’. Significant others face major responsibilities as informal carers, an unrecognised role, which comes with major psychological burdens that significantly impact mental health and well‐being. Research has shown the presence of a cluster of negative mental health outcomes in 34.7% of significant others of ICU patients (McPeake et al. 2016). This cluster of symptoms is termed ‘Post‐intensive care syndrome‐family’ (PICS‐F), and the symptoms can be debilitating (McPeake et al. 2016; Tejero‐Aranguren et al. 2024). The presence of PICS‐F has not been explored in significant others of severe burn injury patients, where the presence and impact on roles may be heightened due to prolonged hospitalisation, disability, disfigurement and challenging mental health outcomes experienced by the patient (Heidari Gorji et al. 2023).

In the Australian context, it is estimated that approximately 1 in 8 Australians are informal carers, meaning approximately 2.7 million (12% of Australians) act in this role often beyond a full‐time capacity (Sarris et al. 2020). It can be surmised that with severe burn injury resulting in prolonged recovery and at times disability, significant others may become ‘forgotten carers’ required to take on the carer role, thereby impacting their own financial security (Tejero‐Aranguren et al. 2024). While research on significant others' experiences is limited, the focus has been on trauma and the long‐term impact of severe burn injury, yet little is known of their experiences in the acute phase (ICU) (Heidari Gorji et al. 2023). Therefore, this research is imperative to capture their experiences in the acute phase and the impact of this life‐altering event on their role within the life of the person with the severe burn injury.

## 3 The Study

### 3.1 Aim

The aim of this paper was to explore how severe burn injury impacts the significant other and their changed role within the injured person's life. This paper is drawn from a larger qualitative study exploring the experiences of significant others of patients with severe burn injuries in the ICU. Other aspects of the significant others' experiences are conceptually different and so have been published elsewhere (Flannery et al. 2024).

## 4 Method

### 4.1 Design

This study used a Narrative Inquiry approach to capture the experiences of significant others. Narrative inquiry is a method that allows participants to share their story of a particular event and attribute meaning to their experience (Polkinghorne 1995). To allow participants to structure and deliver their story in a way that makes sense to them, semi‐structured interviews were used to collect data as participants control the narrative (Polkinghorne 1995). Frank (2013) describes participants' stories as unfinished, and with parts borrowed from the narratives of other people involved (e.g., healthcare providers, family). The story continues to evolve as the experience is ongoing for the participant, and so they continue to contextualise and reform their experience (Frank 2013). The consolidated criteria for reporting qualitative research (COREQ) reporting guidelines were used to guide reporting (Tong et al. 2007) (Data S1).

### 4.2 Participants

The study was conducted in two adult major burns receiving hospitals in New South Wales, Australia. Participants were included if they were a significant other of an adult patient with severe burn injury and were 18 years of age or over. The term ‘significant other’ refers to a person who provides emotional support to the patient and is not a paid carer (Risson et al. 2023; Schimmenti et al. 2020). Participants could be a partner, spouse, child, parent or friend of the patient, and there could be more than one person who felt they filled this role in the patient's life. The patient was considered to have severe burn injury if they required Intensive Care Unit (ICU) admission, intubation and mechanical ventilation. Paediatric patients were not included in this study, as capturing the complex emotions and legal ramifications for intentional burns inflicted on children, burns resulting from neglect, as well as parental feelings associated with blame and guilt were not the aims of this research.

Potential participants were approached by a Social Worker or Clinical Nurse Consultant who worked within the participating ICUs. The Social Worker and Clinical Nurse Consultant were both ideally positioned, and each had over 15 years' experience working with patients with severe burn injury. When the Social Worker and Clinical Nurse Consultant deemed it was appropriate to approach potential participants, they explained the research to the significant other and provided a Participant Information Sheet (PIS). The PIS explained the study, including the right to decline to participate, and the support services they could contact. Potential participants were asked to return the signed consent if they wished to participate and gave permission for the researcher (E.F.) to be given their contact details. The concept of information power guided the sample size determination (Malterud et al. 2016).

### 4.3 Data Collection

The research team (L.M.R., K.P., G.M. and E.H.) is comprised of all Registered Nurse Academics. Four members of the research team (L.M.R., K.P., G.M., E.H.) hold PhDs and are expert qualitative researchers with a wealth of experience in research involving participants who have experienced traumatic events. The first author (E.F.) is a female PhD candidate who had previously worked in one of the research settings and has worked for more than 20 years with patients with severe burn injury and their significant others.

Data were collected during 2021–2022, a period marked by varying COVID‐19 restrictions imposed on Australian hospitals. This meant that participants were interviewed either via video‐conferencing or telephone (Flannery, Peters, et al. 2022). Participants were asked to tell their story as they chose to tell it, in response to semi‐structured interview questions, such as ‘Can you tell me the story of your loved one's burn injury?’. Questions were developed based on the literature and the researchers' clinical expertise. They were deliberately broad to capture and enhance storytelling. The researchers frequently reviewed the questions based on data collected, although no significant changes were made as it captured comprehensive participant stories. Through this process, the research team ensured the research aims were addressed in the questions and that the narrative inquiry methodology was adhered to.

Interviews lasted from 25 to 92 min, with an average of 50 min and were audio‐recorded and transcribed verbatim. While participants were offered the opportunity to review their transcripts, none accepted.

### 4.4 Data Analysis

Data were analysed using narrative analysis (Polkinghorne 1995). Polkinghorne (1995) utilises an approach of creating common threads from the stories, while recognising that each participant's story will have unique aspects. This aligns well with this research, as significant others experienced their loved one having a severe burn injury, but individual responses and experiences were unique while still containing commonalities.

Polkinghorne (1995) identifies the importance of the researcher immersing themselves in stories and through this process, the dominant stories emerge. While Frank (2013) identifies the researcher as being present and bearing witness to the participant's story, it is important that the emergence of dominant stories occurs through this process of sitting with the transcripts, listening and re‐listening for the concepts which are repeated and given emphasis by participants. This ensured the threads were truly representative of the participants' experiences. This process of checking and re‐checking allowed the researcher to then organise data into a meaningful plot. Where threads did not appear across the participants' experiences, they were considered individual responses, rather than threads. Components that did not contribute to answering the research question were not included in the final story.

Through this process the participants' stories were presented in a logical and meaningful way, which conveyed the unfinished stories of their experiences. The research team, who were experienced qualitative researchers, also reviewed the transcripts and threads to ensure the researcher had correctly identified these commonalities and connections, adding clarity and depth in the final presentation of the stories.

### 4.5 Ethical Considerations

Ethics approval was obtained from the hospital and University Human Research Ethics committees (HREC/18/CRGH/267). Participants were not approached by the researcher until they were given a verbal explanation of the research, had an opportunity to read the PIS and had decided to participate. To ensure credibility in the research, the primary researcher adopted a ‘hands off’ approach to participant recruitment in that no participant was approached by the researcher directly, rather through the Social Worker or Clinical Nurse Consultant, thereby removing any coercive effect (Shenton 2004).

Once participants were contacted and read the PIS, the researcher gave them an opportunity to ask any questions prior to giving their consent. All participants signed and returned their consent form via email prior to interviews. As interviews were conducted in the respective homes of the researcher and participant due to COVID‐19 restrictions, a mutually convenient time was decided upon to ensure minimal interruption and safeguard privacy. To further protect privacy and confidentiality, all names were changed to pseudonyms in the final analysis.

### 4.6 Rigour

To ensure credibility, the first author used reflexive journaling (Cypress 2017). This allowed the researcher to identify any assumptions, preconceptions and biases throughout the data collection process. The reflexive journaling process enabled the identification and examination of these influencing factors, fostering self‐awareness in the researcher and allowing for a more objective stance in the research process (Dodgson 2019). The journals were then discussed with the research team, further allowing these experienced qualitative researchers to provide feedback to the PhD candidate (Flannery et al. 2023).

The reflexive journaling process also allowed for peer debriefing to occur, which is another crucial component in ensuring credibility (Dodgson 2019). Peer review is an essential component in the reflexive journaling process, as it also allows for alternate perspectives to be considered; it can help validate researcher perspectives and decrease the potential for biases to impact research (Cypress 2017; Dodgson 2019). Reflexive journaling and peer review were crucial in not only acting as a mechanism for the research team to oversee the data collection process (Dodgson 2019) but also allowed the research team to identify potential vicarious trauma or distress occurring for the first author while interviewing trauma survivors (Flannery et al. 2023). The frequent review process also allowed the team to identify when no new information arose and data redundancy had been reached.

## 5 Findings

Participants were 17 significant others, of whom 13 identified as female and four as male. Five participants were a spouse, two were a sibling, four were a child and six were the parent of the patient with a severe burn injury. All participants had experienced their loved one having a severe burn injury and were faced with complex changes to their pre‐burn relationship with their loved one. However, while the significant others all experienced the changes to relationships and roles, their experiences were different. The findings included three threads, namely, (1) necessary changes in the face of catastrophe, (2) contemplating the future as a carer and (3) lives and relationships redefined. As the participants' stories are unfinished, these threads are not discreet and may cross over as the story unfolds.

The first thread, necessary changes in the face of catastrophe, highlights the practical and immediate actions taken by the significant others and the essential shift in their roles to support the patient with severe burn injury. Actions taken by significant others ensured the patient was given pain relief and that a range of other physical and psychological support needs were met. This thread revealed participants' self‐neglect, during this period of intense trauma. Significant others often prioritised the patient's wellbeing, sometimes to the extent of depriving themselves of their basic needs–such as eating and sleeping—as their sole focus was on guaranteeing the patient's needs were met. The second thread, contemplating the future as a carer, describes the confronting process of the significant other being required to take on a carer role. As severe burn injury was a sudden, unexpected event, significant others were unprepared for their new role and had to consider things such as their own careers, lifestyle and the impact on other family members, such as children. The final thread, lives and relationships redefined, showcases the impact severe burn injury has on the longer‐term relationship of the significant other and the patient, and the subsequent redefining of their roles within the relationship.

### 5.1 Necessary Changes in the Face of Catastrophe

Participants identified that they felt compelled to fulfil new roles when their loved one was hospitalised with a severe burn injury. After the burn injury, participants took on an advocacy role, ensuring the patient's basic needs were met. Many linked this to their loved one being unable to complete activities of daily living because of their burn injury or associated pain. There were a range of roles depending on the severity of the burn injury and the stage of injury and recovery. Nicole's son had sustained burn injuries to his face and arms and was unable to attend to his activities of daily living.

I would feed him. He was totally bandaged like a mummy, so I needed to make sure he was being fed. Nicole

Similarly, John, whose wife sustained severe burn injuries to most of her body in a house fire, described his need to advocate to ensure she was being cared for, and that her medical needs were addressed. John also identified the difficulties he faced processing the trauma, and as a result, his inability to control his feelings of frustration.

I can't leave her like that…they've taken away a piece of bone… surely somebody needs to fix this? I was carrying on a bit. I needed to make sure they fixed it somehow. John

Nicole also recounted the need to ensure her son's medical needs were managed and expressed the pressure she felt to be present to check her son received appropriate and timely care. Similar to John, Nicole experienced frustration if she believed these needs were not being met, and explained that she felt compelled to monitor the situation.

I would get frustrated when they didn't give him pain relief straight away. I felt better when I was there, and I could make sure he got it quickly. Like sometimes if I wasn't there maybe he couldn't push the buzzer easily and it would delay it. Nicole

All participants expressed their fears for how the patient, their loved one, would cope with the aftermath of severe burn injury. They all expressed the importance of psychological support for the patient. This was also to ameliorate their fears that the patient would incur negative mental health outcomes in relation to the burn injury and the associated potential disability and disfigurement.

While participants acknowledged the importance of their loved one's needs being met, they often neglected their own physical and psychological needs. This shift in the relationship marked the beginning of adapting to a carer role, where the significant other put the needs of the patient first. Nicole describes the importance of being present to provide support to her son, while neglecting her own needs:

I needed to be there (in ICU) to just provide support. I thought he might get flashbacks…and hearing my voice would help him know he was ok. I was beyond exhausted really, but I knew I just needed to be there. Nicole

Similarly, Harrison described needing to be present for his wife, while neglecting his own physical needs.

I'd be there as much as they let me, once I left there (the hospital), at night I'd stop the car, the first few days, on the side of the road and just try to decompress or be with my thoughts while I wasn't putting on a front for her. I didn't eat, or shower, or sleep to be honest, for about the first 4 days. Harrison

As further evidence of this, all participants described that they were grateful to be able to share their story of their loved one's severe burn injury. Participants disclosed that they had never shared the entire story of their experiences, prior to their interview. All reported that sharing their stories was an important process, despite never having sought formal support.

Participants all acknowledged their loved one's burn injury was one of the most traumatic moments of their life, as Kim conveyed it was ‘*a catastrophe’*. Interestingly, while recognising this, participants did not seek formal psychological support for themselves; rather, they all reiterated the importance of mental health support for the patient. The experience also led participants to isolate themselves from the external support of family and friends, often in an attempt to shield loved ones from the trauma. These decisions effectively intensified personal psychological harm, as significant others isolated themselves and prioritised the mental health support needs of the patient. This is evident in Kim's response when asked if she accessed support.

For him (her husband), his psychologist—he's got a psychologist, a trauma psychologist that he sees which is important. I think I sat in on one of his sessions once just because I turned up and he said to come in. I didn't seek any help, and I don't see anyone. The focus is on him—it was ‘his’ horrible burn. Kim

Both Harrison and Hayley also disclosed that they did not pursue the psychological support that was offered, instead acknowledging the importance of psychological support for the patient.

Honestly, no. I just sort of pushed everything to the side. I thought my problems are minimal compared to what (my wife) she's dealing with. Harrison

No, you just kind of keep moving. If it's not my husband, then it's obviously just myself in my head. Always counselling myself. Yeah, I don't feel that I am really impacted by it. As I said, I've always felt I've had to take that role, and be the stronger person. I really haven't, yeah, I really would feel bad for him. Hayley

John also did not access support, even though he openly acknowledged that he was not coping. He described that when people asked how he was feeling, he minimised the gravity of the situation and described his experiences of not accessing emotional support.

I've talked to one or two people passing through or whatnot, and just quite factually frankly, I don't really get much out. Really, I've been just a wreck in myself, a complete wreck. I cry a lot. I try to focus on helping her (his wife) get better. John

### 5.2 Contemplating the Future as a Carer

As the patients were intubated and critically ill, participants understood the importance of advocating and, at times, making decisions for the patient when they were unable to do this for themselves. During this time, participants began to contemplate their future as a carer, possibly for an extended period. This often impacted many aspects of their own life and further solidified changes to their role resulting from the injury. For Kylie, this presented a challenging and unexpected change in her life, considering she would be her father's carer.

My biggest worry was probably how…I guess this sounds bad, but how that impacts me when he comes outta hospital? I am only 21. Do they (healthcare providers) expect me to be his full‐time carer? Kylie

While Kylie's story indicates an unexpected role change to being a carer for her father, other participants also described facing difficulties with his changing role, and the ramifications it may have on their future. The carer's role may include wound dressings, monitoring adherence to treatment plans and transporting their loved one to appointments, all of which can place a burden on the significant other and disrupt previous routines. Like Kylie, John also identified uncertainty and fear associated with taking on this new role and responsibility.

there are things she's asked and I don't know the answers…the nature of the injuries, it will take a long time. There's no escaping it, she might…we might…never be the same again. John

Facing difficult choices and increasing responsibility was also identified by Belinda when she became her husband's carer. Belinda shared her new responsibilities and the fears she had about this role.

…he has some exposed bone. I have to keep a close eye on him for signs of infection… Belinda

The ongoing impacts on the significant other's ability to work and, consequently, their financial stability were also noted. Belinda explained that the future was uncertain.

It impacted my work a lot. I'm on casual hours…I just get paid when I'm there. So figuring out how we're gonna pay the bills and all that sort of stuff is stressful. He can't work now, so I'm not really sure what will happen long term. Belinda

Similarly, John also identified increased responsibility in the carer role, and the sense of an undefined future. This was a commonality among the stories in that there was this sense of an uncertain future as a direct result of the severe burn injury and the long‐term recovery process.

At the moment I manage to work to manage most things on my own for her, like her wounds and making sure she does the exercises. I could see that this event, these injuries were quite serious, and would have serious impacts on her life, on my life and maybe permanently. John

### 5.3 Lives and Relationships Redefined

As a result of considerable changes to their roles within their loved one's life, participants and their loved ones were faced with redefining their relationship. Carol identified the ramifications of being a carer to her husband and the profound impact this had on their relationship.

I didn't realise how much it had affected me, but I couldn't fathom a physical intimate relationship with him. He wanted me to be his wife, but I was now his carer. I felt like I couldn't do both‐ it was a really weird feeling. He said “Well is it me?” Do I look ugly? Carol.

Similarly, Belinda shared how taking on the carer role had changed her relationship with her husband. She revealed her need to maintain a carer role, as well as a sense of powerlessness, in potentially losing her carer role as her husband moved towards regaining his independence.

I got really anxious because I had become sort of like a carer role. I was hoping, well, when he went to get his driver's licence, I was hoping they wouldn't give it to him. Belinda

Participants were faced with the complexities of relationship impacts and the redefinition of roles within their loved one's life. As they had already taken on the role of carer, the tendency not to burden their loved one or share their concerns also served to potentially create distance and new pressures within relationships. This is evident in John's story when he disclosed how he prioritised his wife's health over his own by concealing his own health issues.

I've got my own medical issues I haven't dealt with yet, I put them on hold. Now that she's home I should probably follow up, but I am still trying to get her better‐ she is the priority. I actually haven't said anything to her about what's going on‐ I tend to not really tell her problems that she doesn't need to know about, she's got enough to deal with. John

As a result of the severe burn injury, all participants experienced changes in their relationships. All participants were involved in discussions with healthcare providers about their loved one and were required to advocate for them despite experiencing trauma themselves. All participants were required to disclose information to family and friends and had to decide on the level of detail they provided. Participants' relationship with the patient changed by virtue of them being critically ill. As recovery from severe burn injury is a complex and prolonged process, the significant other had to adapt to a long‐term shift in their relationship with the patient and consider how this new role would affect their own life.

## 6 Discussion

This research revealed the experiences of significant others of patients with severe burn injury. The findings illuminated how participants perceived their role in the patient's life both during the intensive care illness phase and in their life thereafter. The threads identified that the participants defined their role in the ICU as focusing on and supporting the physical and psychological needs of the patient. Concurrent to these threads were the threads of significant others' neglecting their own physical and psychological needs and taking on the role of carer for the patient. In most cases, this had significant impacts on their own physical and emotional well‐being, employment and thus finances and impacted significant others' relationship with the patient. The final thread revealed the long‐term impact of the severe burn injury was a forced redefining of the relationship between significant other and the patient. The sudden unexpected severe burn injury, the consequent trauma, critical illness and if the patient survives, long‐term health consequences left significant others in a position where they were relied on as a carer. As evident in our research, the shift to carer role is complex, and can involve major life decisions and has long‐term impacts on the lives of the significant other and the patient with severe burn injury. For some, this will have implications on their ability to work, thereby having a flow on effect to their finances and overall mental health and well‐being.

Previous research has supported the concept of significant others of patients with severe burn injury having an influential role in supporting both the physical and psychological needs of the patient (Bäckström et al. 2019; Flannery et al. 2024). The experience of trauma is understood to be a consequence of severe burn injury for both patients and their significant other (Boersma‐van Dam et al. 2022). Significant others' are reported as being at risk of vicarious trauma, or ‘secondary traumatic stress’ related to exposure to the patient's suffering (Forkey et al. 2023), which can ultimately lead to the development of posttraumatic stress disorder (PTSD) and negatively impacting long‐term health and well‐being (Boersma‐van Dam et al. 2022). Interestingly, Heidari Gorji et al. (2023) reported that significant others identified their lowest need was to be provided with comfort, despite the extreme trauma they were experiencing, which resonated in our research, with participants revealing they declined offers of psychological support. In Australian research, Gullick et al. (2014) identified that the patient and significant other exist together in the traumatic situation post‐severe burn injury, and the significant other focuses on the needs of the patient. While this serves to protect the patient during the critical illness phase, it may also mark the beginning of the change to and redefining of their relationship going forward.

In Australia, it is estimated that informal carers provide approximately 90% of the personal care and housework for people with long‐term conditions and disability (Sarris et al. 2020). Sundara (2014) identified the complex burden experienced by significant others, as the hospital admission marks the beginning of lifelong burdens such as disability and disfigurement, and reliance on the carer extending well beyond the initial critical illness phase. Consistent with this research, significant others were often relied on to provide wound care, monitor for signs of overall deterioration, provide support throughout rehabilitation, assist with activities of daily living and provide psychological and financial support (Sundara 2014). The paucity of research around the experience of significant others of patients with severe burn injury highlights the importance of our research, which explored the significant other's experience of severe burn injury, the direct impact on their lives and likely lifelong changes to their role and relationship with the severe burn injury survivor.

Our findings resonate with those of Ussher et al. (2011) who described changes in roles when exploring roles and role changes between significant others and patients with cancer. Although participants faced different challenges due to the distinct health condition, they reported that they were required to take on quasi‐medical roles, where they were obligated to monitor wounds and responses to treatment (Ussher et al. 2011). In our research, significant others were also required to fulfil this role for their loved ones with severe burn injury. As severe burn injury has a long recovery time, it remains a necessary component of discharge planning to have the carer take on this role, but it is evident in our findings that it alters their relationship with their loved one. For those in an intimate partner relationship, this means the significant other is required to view their loved one's body objectively, shifting from a position of intimacy and taking the perspective of a healthcare provider (Ussher et al. 2011). While this may be helpful in discharging the patient home and in the healing process of the person with severe burn injury, this may impact intimacy and ultimately weaken bonds within the relationship. It is recommended that further research explore how these changes influence emotional well‐being for both patient and significant other, as the relationship transforms alongside the patient's recovery journey.

In a review on carers of patient survivors in intensive care, van Beusekom et al. (2015) identified loss of employment, subsequent financial strain and increased mental health decline among significant others or ‘informal carers’. This was evident in our research, as carers identified the need to reduce their working hours and, at times, cease work to take on this role for their loved one. As recovery from severe burn injury is prolonged (Bäckström et al. 2019), this can lead to significant financial strain, an uncertain future and ultimately major long‐term life changes for significant others. In Australian research, Sarris et al. (2020) also identified that becoming a carer impacts on the person's ability to work, progress in their career and therefore has financial implications. Losses may also include friendships and social opportunities, as well as increasing isolation from others due to the caregiver role (Sarris et al. 2020). The compounding effect of the trauma of severe burn injury, as well as these stressors in the significant other's life, can impact on coping (Flannery et al. 2024; van Beusekom et al. 2015). One coping strategy identified by significant others in this research was a need to be present and support the person with severe burn injury, often making significant personal sacrifices to meet the perceived needs of their loved one, including not eating, sleeping, accessing mental health support or attending to own health care needs. This coincides with the findings in research by Ussher et al. (2011), who identified that self‐sacrifice led to carers' renegotiation of their position in the relationship, where they were automatically required to sacrifice their own wants and needs to meet the needs of the patient. Subsequently, the communication style also changed between significant other and patient, as meeting the patient's needs became the primary focus.

There is a correlation between increased caregiver burden post critical illness and decline in the patient's health status (Bäckström et al. 2019; Sarris et al. 2020; Tejero‐Aranguren et al. 2024). When the patient's health improves, the experience of caregiver burden decreases. This is an important issue for future research into the experiences of significant others with severe burn injury, as this group of patients can experience disfigurement and disability, and a prolonged recovery with frequent access to healthcare (Bäckström et al. 2019); therefore, likely heightening the caregiver's experience of burden.

While some carers cope with long‐term conditions using strategies such as acceptance, positivity and humour, others may adopt maladaptive coping strategies such as self‐blame, overprotectiveness and helplessness (Sarris et al. 2020). In our research, coping in significant others included both positive and maladaptive aspects, which did impact upon relationships with the patient and could be assumed to continue to evolve during the critical illness and recovery phases. These carers' experiences are not well understood, and further research is required. Capturing these experiences is inherent in identifying support to strengthen positive coping strategies and identify support needs. Further, increased knowledge of these experiences will equip healthcare providers with an informed capacity to support significant others through the trajectory of their experience with severe burn injury, while also continuing to provide care to meet the physical and psychological needs of the patient.

## 7 Implications for Practice

This research provides insight into the major life impact of the severe burn injury for significant others. While the focus of providing expert care for the critically ill patient remains imperative, it is important to gain this understanding of the significant other's experience so their support needs can be met, potentially altering the way they cope during the life‐changing event and beyond. With the immense role that significant others play in the survival and recovery of patients with severe burn injury, supporting their needs becomes an integral component of caring for the patient.

With an understanding of the imperative role of the significant other for the patient, nurses and other healthcare providers can take an initial approach of consultation to ascertain the level at which they can provide this support. It is important to gain this information from the significant other, so that realistic expectations can be acknowledged and appropriate psychological support provided. It is also essential to avoid overburdening the significant other, taking into account other roles in their life, such as being a parent to small children and/or working to financially support themselves and their family. A focus on collaboration between healthcare providers and significant others and setting realistic expectations allows for reciprocal respect and support to develop. This rapport building through support will also allow opportunities for healthcare providers to refer significant others to further support services that match their individual needs.

Taking an approach of consulting and embedding support in healthcare providers' engagement with significant others may also reduce the chance that they will decline psychological support and neglect their own needs. This consultative process can be undertaken in individual psychological support sessions or, when appropriate, could involve a facilitated family meeting. While this allows for roles and boundaries to be defined, it also sets the premise that psychological support is necessary. By consulting with the significant other, healthcare providers can plan and set realistic expectations with consideration of individual circumstances. This would increase the importance of ensuring the support needs of significant others are also recognised and identified, and psychological and physical needs met. This approach also acknowledges the significant other's own trauma experience and aims to support and strengthen coping strategies while identifying areas that require further intervention or support.

## 8 Strengths and Limitations

A strength of this research was the use of semi‐structured interviews, enabling participants to share their story without restrictions. This approach worked well in allowing participants to give rich descriptions, as they were not following a firm or structured questioning format. In the case of personal trauma experiences, it also allowed participants to draw components of their story together, as they deemed relevant. This allowed for the findings to be a true representation of their experiences, without influence or bias.

The interviews for this research were conducted during the COVID‐19 pandemic, which may be considered a limitation, as interviews were not face‐to‐face. While participants were given the option of videoconferencing or telephone interviews, 14 of the 17 participants opted for telephone interviews, meaning the researcher could not observe visual cues during the interviews. Face‐to‐face interviews and videoconferencing allow for researcher observations; however, the use of the telephone and being in one's own home can offer a layer of privacy and comfort, thereby increasing the participants' psychological safety and allowing for fuller explanations.

The research being conducted in two sites can restrict transferability. However, the sites represent the two major adult severe burn injury tertiary referral hospitals in NSW, Australia, therefore strengthening the research reliability and thus transferability. The paucity of previous research also creates a space for further research to capture other influencing factors, such as culture, in the significant others' experiences, where broadened knowledge can further inform practice.

## 9 Conclusion

Significant others are exposed to trauma when a loved one sustains a severe burn injury and they endure the subsequent critical illness and recovery process. They require support, yet often decline it, as they transition to a carer role, and consider their physical and emotional needs as unimportant when compared to the needs of the patient. This transition into a carer role is substantially disruptive and can be long term, with far‐reaching lifelong implications to their work, finances, lifestyle and well‐being, with both roles and relationships transformed in both the immediate crisis and in a more profound lifelong sense.

## Author Contributions

All authors have agreed on the final version and meet at least one of the following criteria (recommended by the ICMJE *): (1) substantial contributions to conception and design, acquisition of data or analysis and interpretation of data; (2) drafting the article or revising it critically for important intellectual content.

## Conflicts of Interest

The authors declare no conflicts of interest.

## Supporting information


**Data S1:** jocn70091‐sup‐0001‐DataS1.pdf.

## Data Availability

The data that support the findings of this study are available on request from the corresponding author. The data are not publicly available due to privacy or ethical restrictions.
